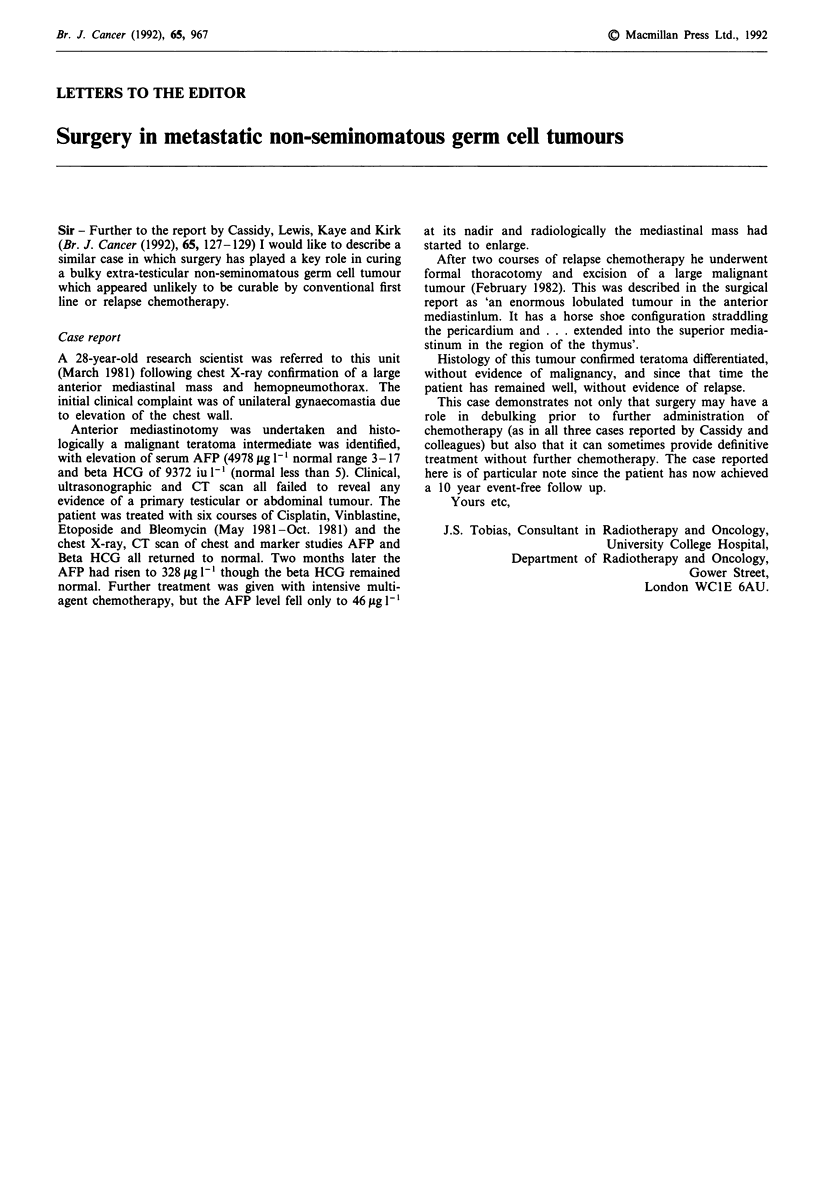# Surgery in metastatic non-seminomatous germ cell tumours.

**DOI:** 10.1038/bjc.1992.202

**Published:** 1992-06

**Authors:** J. S. Tobias


*Br. J. Cancer* (1992), **65**, 967 © Macmillan Press Ltd., 1992

### LETTERS TO THE EDITOR

### Surgery in metastatic non-seminomatous germ cell tumours

**Sir** – Further to the report by Cassidy, Lewis, Kaye and Kirk (*Br. J. Cancer* (1992), **65**, 127–129) I would like to describe a similar case in which surgery has played a key role in curing a bulky extra-testicular non-seminomatous germ cell tumour which appeared unlikely to be curable by conventional first line or relapse chemotherapy.

*Case report*

A 28-year-old research scientist was referred to this unit (March 1981) following chest X-ray confirmation of a large anterior mediastinal mass and hemopneumothorax. The initial clinical complaint was of unilateral gynaecomastia due to elevation of the chest wall.

Anterior mediastinotomy was undertaken and histologically a malignant teratoma intermediate was identified, with elevation of serum AFP (4978 $\mu g\, l^{-1}$ normal range 3–17 and beta HCG of 9372 $iu\, l^{-1}$ (normal less than 5). Clinical, ultrasonographic and CT scan all failed to reveal any evidence of a primary testicular or abdominal tumour. The patient was treated with six courses of Cisplatin, Vinblastine, Etoposide and Bleomycin (May 1981–Oct. 1981) and the chest X-ray, CT scan of chest and marker studies AFP and Beta HCG all returned to normal. Two months later the AFP had risen to 328 $\mu g\, l^{-1}$ though the beta HCG remained normal. Further treatment was given with intensive multi-agent chemotherapy, but the AFP level fell only to 46 $\mu g\, l^{-1}$ at its nadir and radiologically the mediastinal mass had started to enlarge.

After two courses of relapse chemotherapy he underwent formal thoracotomy and excision of a large malignant tumour (February 1982). This was described in the surgical report as 'an enormous lobulated tumour in the anterior mediastinlum. It has a horse shoe configuration straddling the pericardium and . . . extended into the superior mediastinum in the region of the thymus'.

Histology of this tumour confirmed teratoma differentiated, without evidence of malignancy, and since that time the patient has remained well, without evidence of relapse.

This case demonstrates not only that surgery may have a role in debulking prior to further administration of chemotherapy (as in all three cases reported by Cassidy and colleagues) but also that it can sometimes provide definitive treatment without further chemotherapy. The case reported here is of particular note since the patient has now achieved a 10 year event-free follow up.

Yours etc,

J.S. Tobias, Consultant in Radiotherapy and Oncology,
University College Hospital,
Department of Radiotherapy and Oncology,
Gower Street,
London WC1E 6AU.